# Supplementary material for: Dispersive Fourier transform based dual-comb ranging
Source: Nat Commun. 2024 Jun 11;15:4990. doi: 10.1038/s41467-024-49438-z (PMC11167001; doi:10.1038/s41467-024-49438-z)
Supplement: Supplementary file 1 — Supplementary Information [file 41467_2024_49438_MOESM1_ESM.pdf]

Supplementary Information of

# Dispersive Fourier transform based dual-comb ranging


Bing Chang[1,#], Teng Tan[1,2,3,#], Junting Du[1,#], Xinyue He[1], Yupei Liang[1], Zihan Liu[1], Chun Wang[1,4], Handing Xia[4], Zhaohui Wu[4], Jindong Wang[5], Kenneth K. Y. Wong[6], Tao Zhu[5], Lingjiang Kong[2], Bowen Li[1,*], Yunjiang Rao[1,7,*], Baicheng Yao[1,8*]

[1] *Key Laboratory of Optical Fiber Sensing and Communications (Education Ministry of China), University of Electronic Science and Technology of China, Chengdu 611731, China.*

[2] *School of Information and Communication Engineering, University of Electronic Science and Technology of China, Chengdu 611731, China.*

[3] *Institute of Electronic and Information Engineering of UESTC, Guangdong, 523808, China.*

[4] *Research Center of Laser Fusion, China Academic of Engineering Physics, Mianyang 621900, China.*

[5] *Key Laboratory of Optoelectronic Technology & Systems (Education Ministry of China), Chongqing University, Chongqing 400044, China.*

[6] *Department of Electrical and Electronic Engineering, University of Hong Kong, Pokfulam Road, Hong Kong SAR 990777, China.*

[7] *Research Centre for Optical Fiber Sensing, Zhejiang Laboratory; Hangzhou, 310000, China.*

[8] *Engineering Center of Integrated Optoelectronic & Radio Meta-chips, University of Electronic Science and Technology, Chengdu 611731, China.*

**These authors contributed equally:** Bing Chang, Teng Tan, Junting Du

**Corresponding authors:** yaobaicheng@uestc.edu.cn; bowen.li@uestc.edu.cn; yjrao@uestc.edu.cn


**This Supplementary Information consists of the following sections:**


S1. Theoretical analysis

S2. Characterization of the fiber dual-comb source

S3. Extended measurements

Supplementary Figures S1-S12

Supplementary Table S1-S2

Supplementary References S1-S18

## Supplementary note S1: Theoretical analysis

***S1.1 Dual-comb based time-of-flight ranging***

Dual-comb ranging based on the time-of-flight (TOF) method mainly uses the vernier effect of the two pulse trains with a minor repetition rate difference [s1], one comb is used as the ruler (local comb), and the other comb is divided into two parts: a probe comb and a reference comb, they are together called signal comb [s2, s3]. **Fig. S1** shows the schematic diagram of dual-comb vernier effect in time domain. Assuming that the repetition rates of signal comb and local comb are $f_{r1}$ and $f_{r2}$ respectively, the corresponding pulse periods are $T_1$=1/ $f_{r1}$ and $T_2$=1/ $f_{r2}$, and thus the repetition rate difference is $\Delta f_r = |f_{r1} - f_{r2}|$. Here, we mainly discuss the case that $f_{r1}$ is smaller than $f_{r2}$ ($T_1 > T_2$) for instance.

In one complete update period ($T_{update}=1/\Delta f_r$) of a dual-comb interferometry, determined by the repetition rate difference between the two combs, the local comb will encounter the reference comb and the probe comb in turn. At this point, one can always find the case where two adjacent local pulses are sandwiched between two adjacent signal pulses, as shown in the dotted box at the bottom of **Fig. S1**, which produce four types of pulse-to-pulse time intervals ($\tau_1$~$\tau_4$), corresponding to the interval between pulses **i** and **iii** ($\tau_1$), pulses **ii** and **iv** ($\tau_2$), pulses **v** and **vii** ($\tau_3$), and pulses **vi** and **viii** ($\tau_4$). Obviously, they meet the following relationship:

$$\tau_1 + \tau_2 = \tau_3 + \tau_4 = \Delta T_r = |T_1 - T_2| \tag{1}$$

When we regard the time of pulse **i** as the benchmark ($t_i$ = 0), the time of pulse **ii**~ **viii** can be written as follows:

$t_{ii} = T_1$, $t_{iii} = \tau_1$, $t_{iv} = \tau_1+T_2$, $t_v = t_{tof} + (N\text{-}1)\, T_1$, $t_{vi} = t_{tof} + N\times T_1$, $t_{vii} = t_{iii} + N\times T_2 = \tau_1 + N\times T_2$, $t_{viii} = \tau_1 + (N+1) \times T_2$.

Here $t_{tof}$ is the time of flight. Since $t_{vii} - t_v = \tau_3$, we can obtain the pulse-to-pulse delay $t_{tof}$ between the probe comb and the reference comb by combining the above time expressions:

$$\begin{aligned} t_{tof} &= NT_2 - (N-1)T_1 + \tau_1 - \tau_3 \\ &= T_1 - N(T_1 - T_2) + \tau_1 - \tau_3 \\ &= T_1 - N\Delta T_r + \tau_1 - \tau_3 \end{aligned} \tag{2}$$

Wherein, the integer $N$ can be obtained by:

$$N = (t_{vii} - t_{iii}) / T_2 \tag{3}$$

Then the distance can be calculated by $L_D = c\cdot t_{tof}/2n$, $n$ denotes the refractive index of the light transmitting medium. For instance, in air, $n$ = 1.

It is worth mentioning that although we only use $\tau_1$ and $\tau_3$ when calculating $t_{tof}$, $\tau_2$ and $\tau_4$ are equally important, because it can help us judge the relative position of the signal comb pulse and the

local comb pulse, based on **Eq. (1)**.

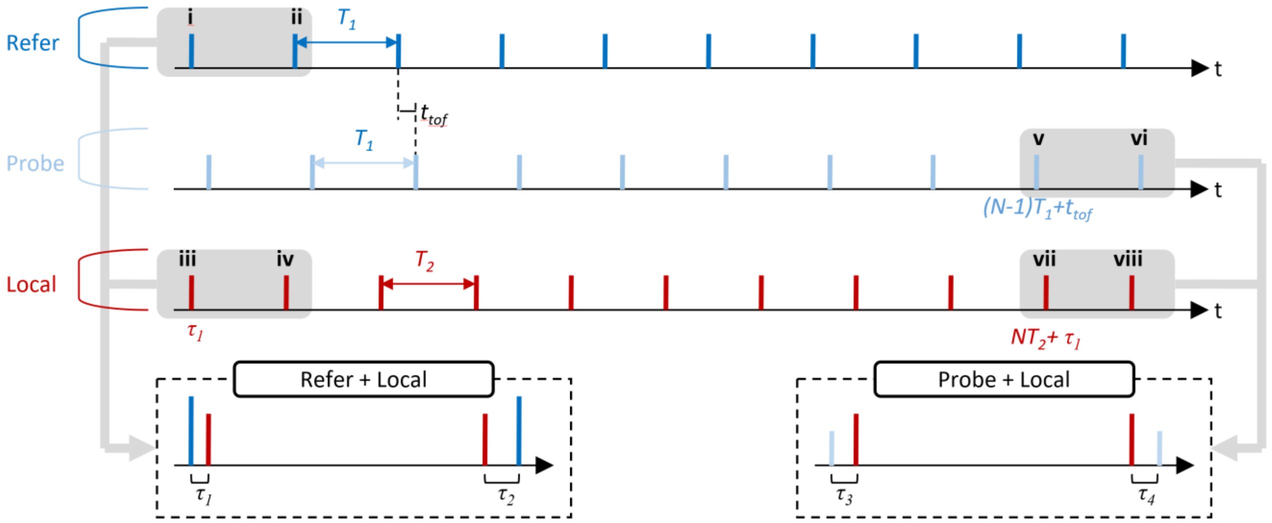


**Fig. S1. Schematic diagram of dual-comb pulse interference.** Here the dark blue pulses show the reference comb, light blue pulses show the probe comb, they have the same period $T_1$. The red pulses show the local comb, which has a period $T_2$. Dual-comb interferences only appear when two pulses overlap.

### *S1.2 Dispersive Fourier Transformation*

According to **Eq.** (**2**), the detection accuracy of the time-of-flight of the signal comb pulse depends on the temporal demodulation accuracy of $\tau_1$ and $\tau_3$, which determines the detection accuracy the dual-comb ranging. Commonly, limited by the bandwidth of a photodetector (up to tens of gigahertz), it is impossible to distinguish two pulses whose time interval is in the order of sub picoseconds. On the other hand, the dispersive Fourier transform (DFT) provides a unique tool for visualizing and analyzing ultrafast time-domain signals [s4], which can effectively satisfy the precise measurement requirement of pulse intervals in ranging applications.

Starting from the classical electromagnetic theory, we define $E_1$ to represent the output electric field of a soliton pulse, whose spectrum can be written as:

$$E_1(\omega) = \mathcal{F}\{E_1(t)\} = |E_1(\omega)| \exp(i\theta(\omega)) \tag{4}$$

Herein $\mathcal{F}$ illustrates the Fourier transform, $E_1(\omega)$ and $\theta(\omega)$ denote the magnitude and phase of the soliton spectrum, respectively. The chirp of the pulse can be written as $\theta'(\omega) = d\theta(\omega)/d\omega$, which equals zero when there is no chirp. Now consider a dispersive element with a length $L$. Its transfer function of linear group-delay dispersion (GDD) is in the following form [s5]:

$$H(\omega) = \exp(i\phi(\omega)) \tag{5}$$

Herein $\phi(\omega)$ is the phase profile and it can be Taylor-expanded as:

$$\phi(\omega) = -\sum_{m=0}^{\infty} \frac{\beta_m L}{m!} \omega^m = -\beta_0 L - \beta_1 L\omega - \frac{1}{2}\beta_2 L\omega^2 - \cdots \tag{6}$$

Then the group delay can be written as:

$$\tau(\omega) = -\frac{d\phi(\omega)}{d\omega} = \sum_{m=1}^{\infty} \frac{\beta_m L}{(m-1)!} \omega^{m-1} \tag{7}$$

Here $\beta_m$ is the m$^{th}$-order coefficient of the expansion, where the group velocity is $1/\beta_1$ while the group velocity dispersion (GVD) is $\beta_2$. Assuming without loss of generality, that means $\beta_0 = 0$, the electric field of a transformation-limited pulse (without chirp) passing through a large GVD element could be deduced as:

$$\begin{aligned} E_2(t) &= \mathcal{F}^{-1}\left\{E_1(\omega)\cdot H(\omega)\right\} \\ &= \frac{1}{2\pi}\int_{-\infty}^{+\infty} \left|E_1(\omega)\right| \exp[i(\theta(\omega)+\phi(\omega)+\omega t)]d\omega \\ &= \frac{1}{2\pi}\int_{-\infty}^{+\infty} \left|E_1(\omega)\right| \exp[i(\theta(\omega)+\hat{\phi}(\omega)-\beta_1 L\omega+\omega t)]d\omega \\ &= \frac{1}{2\pi}\int_{-\infty}^{+\infty} \left|E_1(\omega)\right| \exp[i(\theta(\omega)+\hat{\phi}(\omega)+\omega T)]d\omega \end{aligned} \tag{8}$$

Herein, $\hat{\phi}(\omega)$ represents the retarded phase profile,

$$\hat{\phi}(\omega) = \phi(\omega) - \beta_1 L\omega = -\sum_{m=2}^{\infty} \frac{\beta_m L}{m!} \omega^m \tag{9}$$

corresponding to a retarded group delay

$$\hat{\tau}(\omega) = -\frac{d\hat{\phi}(\omega)}{d\omega} = \sum_{m=2}^{\infty} \frac{\beta_m L}{(m-1)!} \omega^{m-1} \tag{10}$$

Here $T = t - \beta_1 L$ represents the time variable retarded by dispersion element.

Similar to Fraunhofer diffraction, the spectrum of a pulse can be mapped to the time domain when sufficient dispersion satisfies the stationary phase approximation (SPA) [s6, s7]. In the case of linear dispersion, the classic time-stretched dispersion Fourier transform (TS-DFT) can be obtained. So, according to SPA condition, under a large GVD condition, the term $\exp[i(\theta(\omega)+\hat{\phi}(\omega)-\omega T)$ in **Eq. (8)** would oscillate rapidly and cause the integral to vanish, except in a small range around the stationary frequency $\omega_s$.

$$\frac{d}{d\omega}(\theta(\omega)+\hat{\phi}(\omega)+\omega T)\bigg|_{\omega=\omega_s} = \frac{d(\theta(\omega))}{d\omega}\bigg|_{\omega=\omega_s} + \frac{d\hat{\phi}(\omega)}{d\omega}\bigg|_{\omega=\omega_s} + T = 0 \tag{11}$$

Here, $d\theta(\omega)/d\omega$ denotes the initial chirp of the incident pulse, which can be ignored for near-transform-limited pulses. When high-order dispersion is not considered, **Eq. (11)** can be further simplified as:

$$T = \beta_2 L \omega_s \tag{12}$$

Accordingly, in the case that there is only linear dispersion (i.e. group delay is a linear function of optical frequency), the time delay of the frequency component $\omega$ in the pulse is $T$. Therefore, the spectral components in the output pulse are separated, and thus there exists a linear map between frequency and time. When two pulses with a small time delay are simultaneously transmitting in a dispersive element, the two pulses will be co-stretched and overlap each other. Since their frequency components are spread out in time, spectral interference occurs in their overlapping parts. Considering the central moment of the **pulse1** is $t$, and the central moment of the **pulse2** is $t$-$\tau$, the instantaneous frequency of the **pulse1** and the **pulse2** are $f_1 = t/2\pi\beta_2 L$ and $f_1 = (t-\tau)/2\pi\beta_2 L$, respectively. Here $\tau$ is the relative delay of the two pulses. The frequency difference $f_i$ at each moment is [s8]:

$$f_i = |f_1 - f_2| = \frac{\tau}{2\pi|\beta_2|L} \tag{13}$$

As a result, we can obtain the relative delay of the two pulses through the spectral analysis of the interference fringes. When considering higher-order dispersions such as the third order dispersion $\beta_3$, the time delay of the frequency component $\omega$ in the pulse would become more complicated:

$$T = \beta_2 L\omega + \frac{\beta_3 L\omega^2}{2} \tag{14}$$

Then the instantaneous frequency of the **pulse1** and the **pulse2** are:

$$\begin{aligned} f_1 &= \frac{t}{2\pi\beta_2 L} - \frac{\beta_3 L t^2}{4\pi(\beta_2 L)^3} \\ f_2 &= \frac{t-\tau}{2\pi\beta_2 L} - \frac{\beta_3 L(t-\tau)^2}{4\pi(\beta_2 L)^3} \end{aligned} \tag{15}$$

In this case, we can also write the interferometric fringe frequency as:

$$f_i = f_1 - f_2 = \frac{\tau}{2\pi\beta_2 L} + \frac{\beta_3 L[(t-\tau)^2 - t^2]}{4\pi(\beta_2 L)^3} \tag{16}$$

The beating frequency of the above equation can be divided into a beat frequency caused by linear dispersion and another beat frequency caused by the third-order dispersion.

In our experiment, two dispersion-compensating fiber modules (Corning PureForm DCM-D-080-04) were used as the dispersive element with a dispersion parameter of $D$ = 87 ps/nm·km (The total length is 30.39 km) and a dispersion slope of $S$ = 0.025 ps/nm$^2$·km. Correspondingly, $\beta_2$ = 110.94

ps$^2$/km and $\beta_3$ = 0.142 ps$^3$/km can be obtained after simple calculation. It can be seen that the frequency chirp caused by third-order dispersion is much smaller than that of linear dispersion so that it can be ignored.

### *S1.3 Parametric Simulation and Discussion*

Based on **Eq. (13)**, for a pulse delay τ, the frequency of interference fringes depends on the total dispersion of the dispersive element. Therefore, optimization of the total dispersion amount is an important issue in practical experiment. The total dispersion $|\beta_2|L$ influences the effect of the time-stretched DFT by limiting the range of pulse delay which we can measure, and changing the fringe frequency directly. Here, we discuss the relationship between the amount of the total dispersion $|\beta_2|L$ and main parameters of the optical frequency comb ($\Delta\lambda, f_{r1}, f_{r2}, \Delta f_{rep}$) in simulations.

On one hand, $|\beta_2|L$ cannot be too large. According to the DFT, the broadened pulse can have an enlarged temporal width $\delta$,

$$\delta = \frac{2\pi c}{\lambda^2}|\beta_2|L\Delta\lambda \tag{17}$$

Referring the cross-relationship among the stretched pulse width $\delta$, the spectral 3dB bandwidth $\Delta\lambda$, and the dispersion $|\beta_2|L$ of the dispersive element (**Fig. S2a**), in order to avoid pulse aliasing, the stretched pulse width should not exceed the pulse repetition period (the dashed yellow line in **Fig. S2a**), that is:

$$\delta < \min\left[\frac{1}{f_{ri}}\right](i=1,2) \tag{18}$$

Correspondingly, for an optical frequency comb with spectral width of Δλ, the amount of dispersion has a maximum value:

$$|\beta_2|L < \min\left[\frac{\lambda^2}{2\pi c\Delta\lambda f_{ri}}\right](i=1,2) \tag{19}$$

**Fig. S2b** shows the interference fringe frequency $f_i$, versus with the pulse delay $\tau$ and the total dispersion $|\beta_2|L$. According to **Eq. (13)**, $f_i$ is proportional to $\tau$. Meanwhile, $|\beta_2|L$ affects its slope (chirp rate). Besides, in the DFT-enhanced dual-comb ranging system, **Eq. (2)** requires that the maximum detectable pulse delay should be larger than the maximum possible value of $\tau_i$ (i=1,2,3,4), we write:

$$\tau_{\max} > \Delta T_r = |T_1 - T_2| = \left|\frac{f_{r2} - f_{r1}}{f_{r1}f_{r2}}\right| = \frac{|\Delta f_{rep}|}{f_{r1}f_{r2}} \tag{20}$$

On the other hand, the bandwidth limitation of the detector $B_{pd}$ should be considered. In **Fig. S2b**, we plot the case when we cannot detect the fringes with frequencies larger than the photodetector's

bandwidth (the dashed yellow line). Due to the above double limitation (the maximum detectable pulse delay and $B_{pd}$), we estimate a minimum value of the total dispersion:

$$|\beta_2|L > \frac{|\Delta f_{rep}|}{2\pi f_{r1} f_{r2} B_{pd}} \tag{21}$$

Furthermore, when **Eq. (19)** and **Eq. (21)** are satisfied, a larger total dispersion and a wider pulse broadening, would bring more fringes and higher signal-to-noise ratio (SNR) in the spectrum after Fast Fourier transform (FFT). In practice, to obtain a large total dispersion commonly relies on the increment of $L$ (the length of dispersive element), which is usually accompanied by considerable losses. In addition, according to **Eq. (2)**, in the DFT enhanced dual-comb ranging system, the distance measurement result can be expressed as:

$$D = \frac{c}{2n} t_{tof} = \frac{c}{2n}(T_1 - N\Delta T_r + \tau_1 - \tau_3) \tag{22}$$

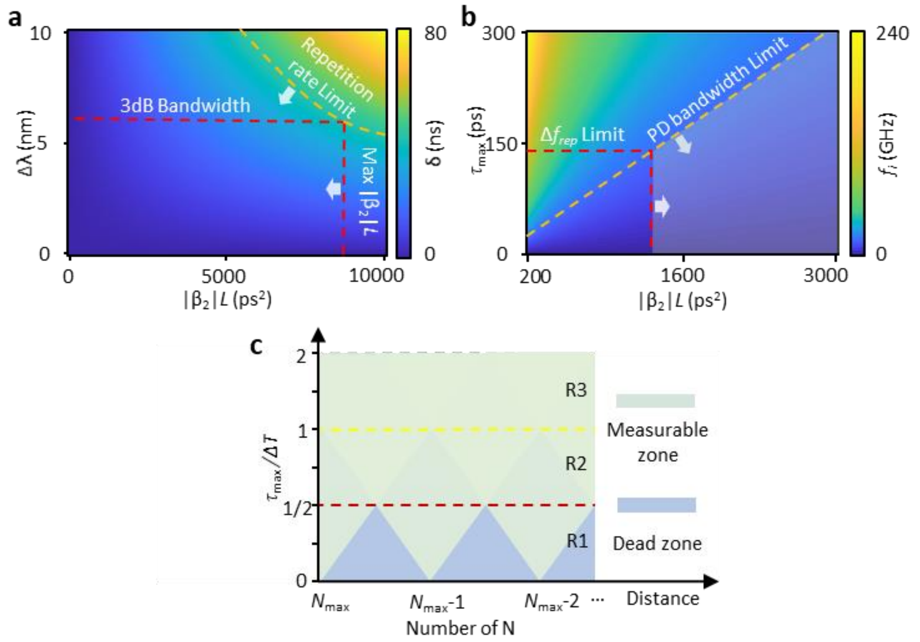


**Fig. S2. Parametric Simulation and Discussion. a.** Simulated parameter space, showing the relationship between $\delta$, $\Delta\lambda$, and $|\beta_2|L$. **b.** Simulated parameter space, showing the relationship between $f_i$, $\tau_{max}$, and $|\beta_2|L$. **c.** The relationship between the ranging distance and $\tau_{max}$.

Since $N$ is an integer, the measurement of distance can be divided into discrete intervals (corresponding to different $N$), and the measurement range of each interval is determined by $\tau_1$-$\tau_3$. Here, we divide the value range of $\tau_1$-$\tau_3$ into three intervals, as shown in **Fig. S2c**. One is $\tau_{max} < \Delta T_r/2$, corresponding to a value range of $\tau_1$-$\tau_3$ is $(-\Delta T_r/2, \Delta T_r/2)$ (**R1**). There are two problems in this case, on

the one hand, we cannot obtain correct $\tau_1$ and $\tau_3$, because we cannot obtain the complete $\tau_1$, $\tau_2$, $\tau_3$, $\tau_4$ through **Eq. (1)**. On the other hand, the distance measurement is discontinuous, which means there is a measurement dead zone (purple part in the **Fig. S2c**). The second case is $\Delta T_r/2 \leq \tau_{max} < \Delta T_r$, corresponding to a value range of $\tau_1$-$\tau_3$ is ($-\Delta T_r$, $\Delta T_r$) (**R2**). In this case, although the distance can be covered continuously, it still does not satisfy the **Eq. (1)**, so that the correct $\tau_1$ and $\tau_3$ cannot be obtained in each frame. The third case is $\tau_{max} > \Delta T_r$ (**R3**), in this case, the measurable distance is continuous and we can obtain $\tau_1$ and $\tau_3$ with certainty for each frame. In our system, we meet the requirements of **R3** by selecting a larger GVD.

# Supplementary Note S2: Characterization of the fiber dual-comb source

### *S2.1 Experimental setup*

The dual-comb fiber mode-locked laser used in the experiment is realized via a hybrid passive mode-locking mechanism, by using the NPR effect and intracavity semiconductor saturable absorber mirror (SESAM). Each fiber laser comb is composed by a 980 nm pump laser diode, a section of 2 m highly-nonlinear erbium doped fiber (HNL-EDF), a SESAM, a section of 6 m single mode fiber (SMF), a squeeze polarization controller (PC), a polarization dependent-optical integrated component (PD-OIC) and a piezoelectric ceramic transducer (PZT), as shown in **Fig. S3a**. The HNL-EDF simultaneously provides rare-earth gain for the laser excitation and high nonlinear gain for nonlinear interactions (e.g. SPM, XPM and SRS). The PD-OIC offers comprehensive functions including 980/1550nm wavelength-division-multiplexing, 80/20 coupling, and polarization-selective-isolation, which can also help to suppress the backward Brillouin scattering. By adjusting the polarization of the light in the cavity, we generate a soliton mode-locked pulse sequence with average output power of 0.5 mW when the pump power was 10 mW.

For fully locking the repetition rates of each fiber comb respectively, we control the cavity length $L_f$ of the fiber loop by using a PZT. **Fig. S3a** also shows the servo feedback control setup for the repetition rate locking. First, the difference between the comb repetition frequency (detected by the photodetector) and an ultra-stable frequency signal (from a standard radio frequency oscillator) is mixed through a frequency mixer (Mini-circuits ZLW-6+), generating a down-converted reference. A low-pass filter (Mini-circuits BLP-5+, DC to 1.9 MHz) is then used to filter out the low-frequency differential signal, which works as the error signal after amplified. Finally, a high-speed servo controller (Vescent D2-125-IP-230) is used to generate a feedback control signal according to the input error signal to drive the voltage controller on the PZT (Core morrow E53.A1K, Input 0~10V, output 0~150V). Due to the electrostriction effect of PZT (maximum diameter expansion 0.38 μm at 150V),

a cavity length stretches of 10 μm can be achieved under full load driving, which corresponds to a repetition frequency locking range of 30 Hz. The controlling accuracy of the feedback loop is 0.003 μm. Since we used two laser combs, two feedback loops are essential. In future, an alternative scheme is using a pair of counter-propagating laser combs, which share the same laser cavity, as **Fig. S3b** demonstrates. Electronic configuration is the same as single comb. In details, due to the bidirectional gain, counter-propagating dual-comb generated in a single cavity is an effective way to simplify the current dual-comb configuration. This can be realized by wavelength multiplexing, polarization multiplexing, or bidirectional mode-locking [s9, s10]. For example, **Fig. S3b** shows the experimental set-up for a bidirectional mode-locked laser comb. By fine tuning the pump power and intra-cavity polarization, frequency combs can be generated from both circulating directions, with repetition rate difference originating from center-wavelength and group-velocity dispersion. Thanks to the common-mode noise cancellation scheme, the repetition rate difference is inherently stable. Therefore, a single set of phase lock loop to lock the repetition rate of either comb is sufficient for dual-comb stabilization.

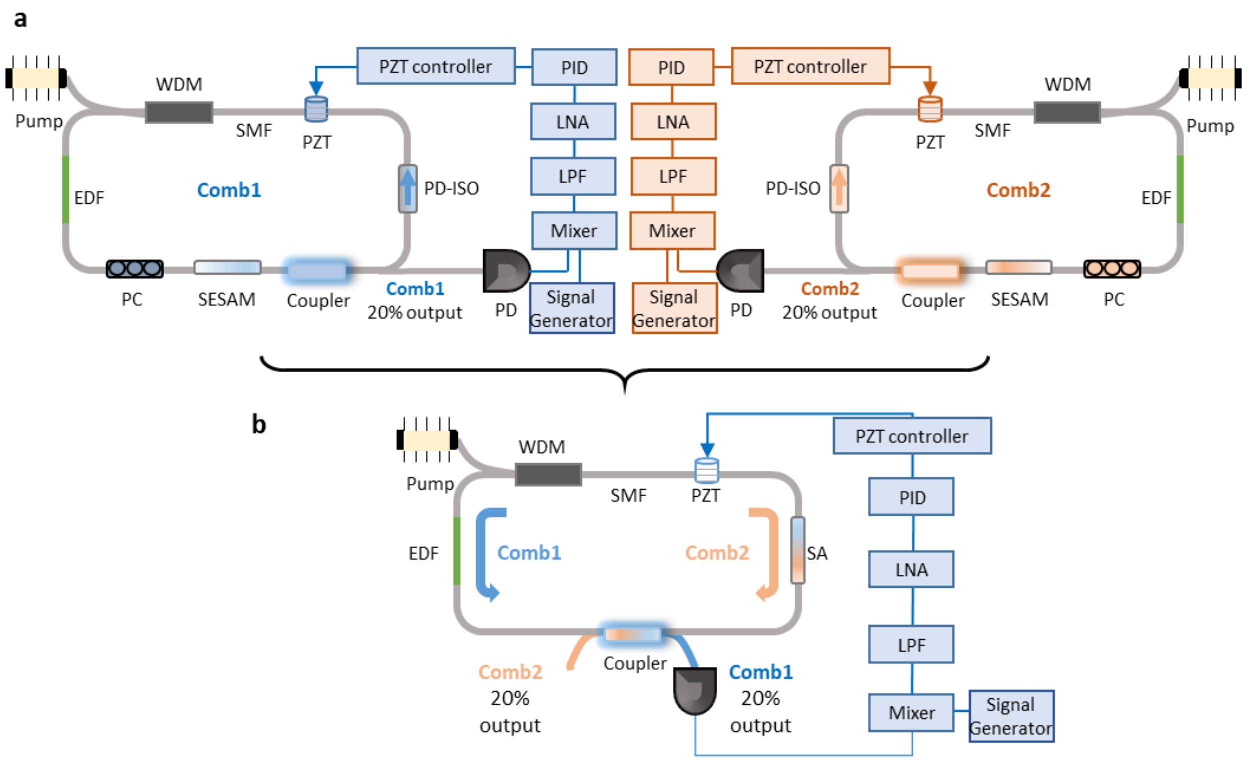


**Fig. S3. Experimental setup of fiber comb generation. a.** Two laser combs and their stabilization setups. **b.** Simplified design based on counter-propagating dual combs generated in one cavity. PZT: piezoelectric ceramics, PID: proportional-integral-derivative controller, LNA: low noise amplifier, LPF: low pass filter, SMF: single mode fiber, PC: polarization controller, EDF: erbium-doped-fiber, WDM: wavelength division multiplexer.

### *S2.2 Dual-comb characterization*

We characterize the output pulses of the signal laser comb and the local laser comb separately. **Fig. S4a** demonstrates the measured optical spectra of the two combs. Their full width at half maximum (FWHM) is about 6.84 nm (0.855 THz) for the local comb and 6.09 nm (0.761 THz) for the signal comb, respectively. The Kelly sideband is 25dB lower than the main peak, which means that the energy of the dispersion wave is low enough compared to the soliton envelope. Using the second harmonic generation (SHG)-based autocorrelation technique, we measure frequency-resolved-optical-gating (FROG) maps of the two soliton pulses (**Fig. S4b**). The retrieved pulse duration of the local and the signal comb is 368 fs and 416 fs, corresponding to time-bandwidth products of 0.315 (local comb) and 0.316 (signal comb), respectively, approaching the Fourier transform limit. In **Fig. S4c**, we show the line-to-line beat notes of the local comb and the signal comb. It verifies that repetition rate of the local comb is 24.55 MHz, while repetition rate of the signal comb is 24.465 MHz. For the first self-beating frequency of each comb, the SNR is higher than 70 dB.

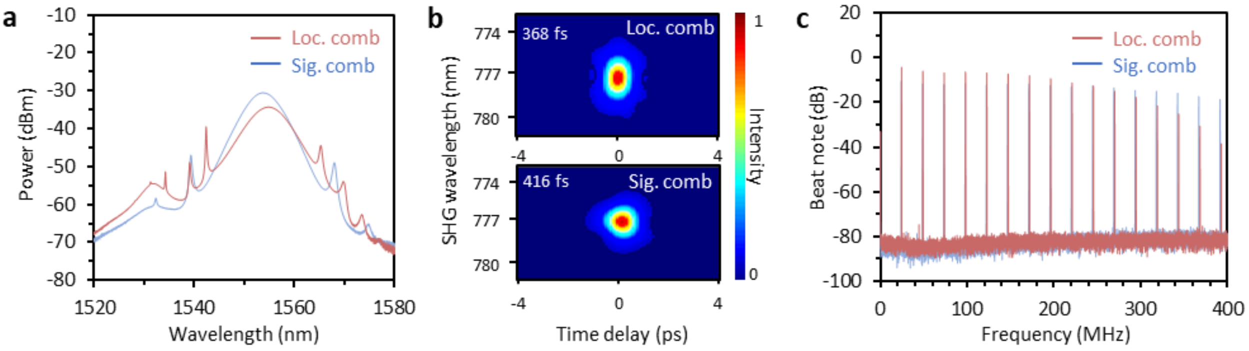


**Fig. S4. Frequency and time characterization. a.** Measured optical spectra of the local comb and the signal comb. **b.** Measured FROG maps of the local comb and the signal comb. **c.** Measured self-beat notes of the local comb and the signal comb.

In electronics, we test the noises of our comb sources. First, we measure the relative intensity noise (RIN) of our comb source in the top panel of **Fig. S5a**. Typically, the RIN of each laser comb is -98 dBc/Hz at 1 Hz offset, -121 dBc/Hz at 1 kHz offset, and < -150 dBc/Hz at 1 MHz offset. In addition, we measure the single sideband phase noise (SSB-PN) by using a Phase Noise Analyzer (Rohde & Schwarz FSWP26) in the bottom panel of **Fig. S5a**. Typically, the SSB-PN of either the signal comb or the local comb (carrier ≈ 24.55 MHz) is down to -75 dB/Hz at 1 Hz offset, -126 dBc/Hz at 1 kHz offset, while -134 dB/Hz at 1 MHz offset. In **Fig. S5b**, we show the measured Allan deviation. When the averaging time is 2.5 ms, the Alan deviation of the two combs reaches the minimum value, telling a number $1.26\times10^{-10}$. In **Fig. S5c**, we show the measured spectrum of the dual-comb beating (first

beating line). First we confirm that the dual comb beats show a difference frequency 85 kHz, it demonstrates a signal-to-noise ratio > 60 dB. There are no excess heterogeneous frequency components in the low-frequency section. Moreover, we also characterize the long-term frequency stability of the two combs in free-running and locked states, respectively. **Fig. S5d** plots the frequency drift of the two combs within 60 s in the free-running (blue) and locked (red) states, respectively. Frequency drift reduced from 6 Hz to 0.25 mHz after locking. In this figure, we also show the stability of the radio frequency reference. Due to the locking operation, performance of the comb sources is mainly limited by the microwave generator.

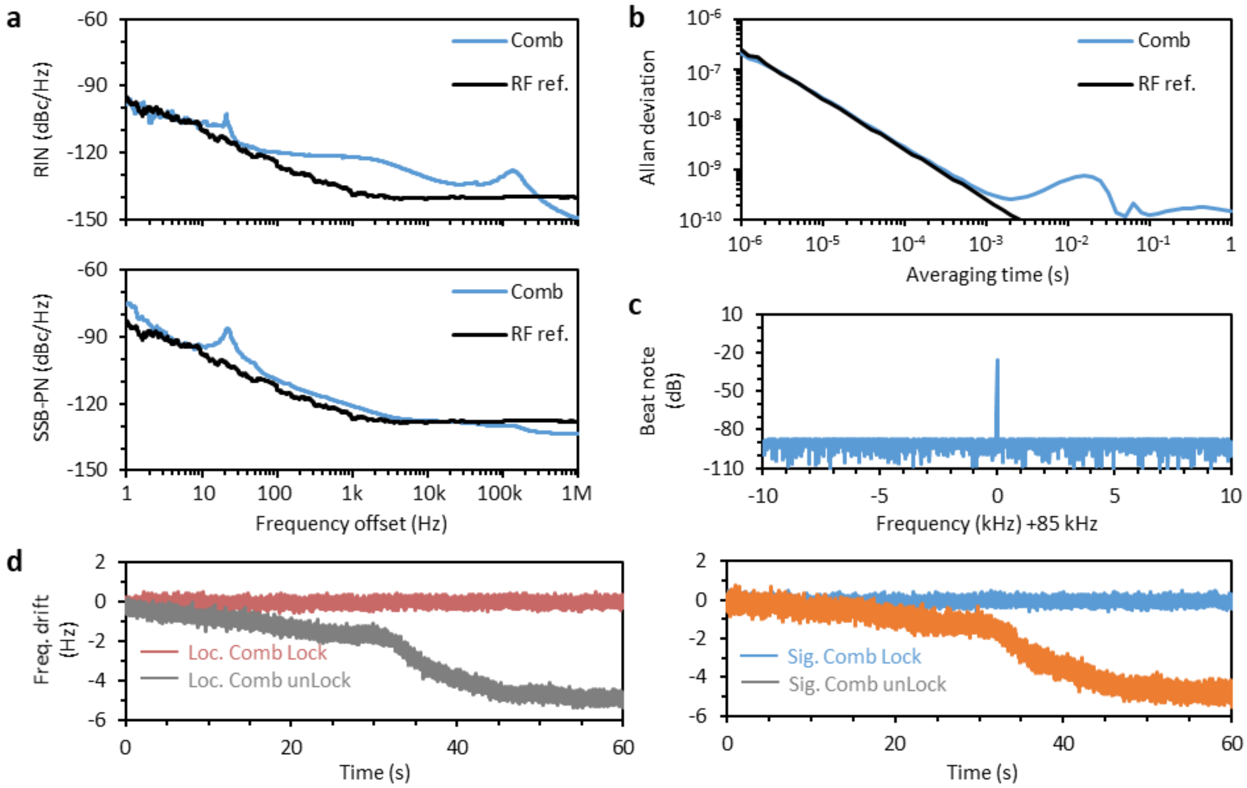


**Fig. S5. Stability characterization. a.** Measured RIN and SSB-PN of our stabilized fiber laser comb. **b.** Allan deviation, it suggests a minimum number at the averaging time of 2.5 ms. **c.** Measured beat note of the dual comb heterodyne (signal and local). **d.** Measured frequency drifting of the local comb and the signal comb. Here the grey curves show the unlock (free running) case.

## Supplementary Note S3: Extended measurements

### *S3.1 DFT characterization*

We use the experimental setup (shown in **Fig. S6a)** to characterize the real-time interferometry based on the DFT. One of the dual combs ($f_{rep}$=24.465MHz) is divided into two paths through a fiber coupler (50:50), one path passes through a motorized delay stage, and the other path passes through a

polarization controller (PC) to maximize modulation depth of the interference fringes. The two paths are then coupled together. The large group velocity dispersion (GVD) is provided by two dispersion-compensating fiber modules (high dispersive fiber, or HDF, Corning PureForm DCM-D-080-04) with a total group delay dispersion (GDD) of 2644 ps/nm. Here, for consistency, the dispersive element is same as in the ranging experiment. Due to the high insertion-loss of the HDF, a low noise erbium-doped fiber amplifier (EDFA) is used to boost the optical power. The interference signal is detected in a photodetector (Newport, Model 1414, 25 GHz bandwidth) and digitized in a high-speed oscilloscope (Tektronix, DPO72004B, 20 GHz bandwidth, 80 GHz sampling rate). **Fig. S6b** shows the temporal waveforms of the output pulses of the mode-locked laser before and after dispersion stretching. The initial pulse width is 368 fs, while the stretched pulse width is 18 ns. By adjusting the fiber delay line, clear interference fringes are observed in the oscilloscope. **Fig. S6c** shows the interferogram when the pulse delay are 8 ps, 16 ps and 80 ps respectively. After Fourier transform, the interference signal demonstrates corresponding fringe frequencies 0.3726GHz, 0.7453GHz and 3.7264GHz. We then characterize the accuracy of the DFT. **Fig. S6d** shows the measured frequencies of the interference fringes, scale with the pulse delays. The result suggests high linearity, the correlation coefficient $R$ of its linear fit is approximately equal to 0.99999. and the slope matches the dispersion medium, and **Fig. S6e** shows the difference between the retrieval pulse separation measured by the DFT and the pre-set value. Uncertainty is in single femtosecond level. The small difference may be caused by the random fluctuation of the fiber link or the noise of Digital acquisition system. Additionally, we monitored 1,000 consecutive measurement sets (total length 2000 s) with identical pulse delays. As depicted in **Fig. S6f**, the measurement error stays within ±1 fs. That means, the ranging error due to the long term instability is below 150 nm for a single shot. Here, the three traces show the cases that we pre-set the delays 36.95ps, 87.00ps, and 137.99ps, respectively. This indicates that by positioning the dispersive fiber in a stable environment, the error induced by its length fluctuations can be essentially mitigated.

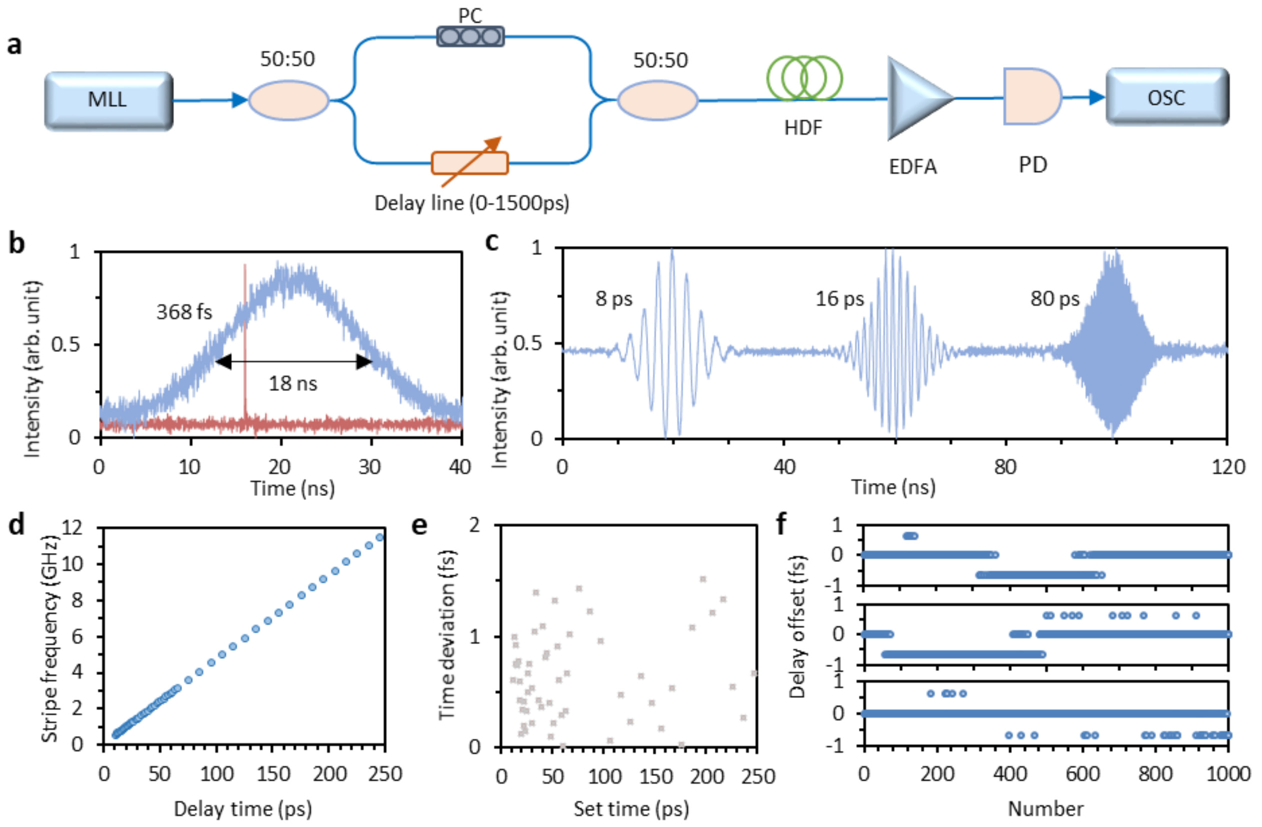


**Fig. S6. Characterization of DFT method. a.** Experimental setup for characterizing the DFT. **b.** The measured pulse before and after dispersion stretching. **c.** Interference fringes for different time delays. **d.** The relationship between the time delay and the frequency of fringes. **e.** The absolute error between DFT retrieval time delay and pre-set time delay. **f.** Statistic measurements under different pulse delays. (Pre-setting time delay: top: 36.95ps; medium: 87.00ps; bottom: 137.99ps).

Compared to the transformation-limited femtosecond pulse, the dispersion-induced linear broadening results in a temporal width extending into tens of nanoseconds, for the pulse after DFT. This broadening allows for higher power amplification in an Erbium-Doped Fiber Amplifier (EDFA) while minimizing nonlinear effects. This feature represents one of the key advantages of our method over the traditional dual-comb ranging schemes that rely on ultrashort pulses. As illustrated in **Fig. S7**, we amplify the same pulse before and after chirp-based broadening. By using the same C-band EDFA, we analyze their spectra. Due to the short duration and high peak power of transformation-limited pulses, their amplification efficiency is relatively low, making such pulses challenging to amplify effectively. For example, when the output power reaches 0 dBm, the spectrum of an ultrafast pulse undergoes significant supercontinuum broadening and deformation, with little increase in peak power. In contrast, the dispersion-stretched pulse retains its spectrum remarkably well even when amplified to 32 dBm. These results highlight the unique advantage of our DFT-based approach in enhancing

pulse energy, rendering it particularly suitable for out-of-lab applications such as long-distance ranging.

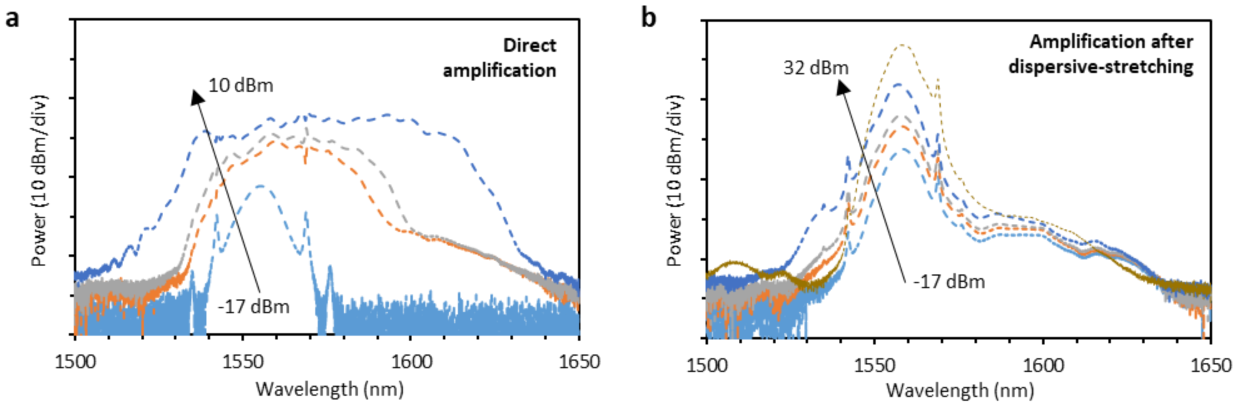


**Fig. S7. Amplified spectra of a pulse before and after dispersive-stretching. a.** Amplified spectra of pulse without chirping. Curves from low to high, output power is -17 dBm, 0 dBm, 3 dBm, 10 dBm. **b.** Amplified spectra of pulse after stretching. Curves from low to high, output power is -17 dBm, 0 dBm, 3 dBm, 10 dBm, 32 dBm.

### *S3.2 Signal processing method*

Now we provide more details about our signal processing method in ranging experiment. The interferograms are detected by an AC-coupled 25-GHz InGaAs photodetector (Model 1414, Newport) and digitized with a high-speed 20-GHz oscilloscope (DPO72004B, Tektronix), commonly we can use the sampling rate at 50 $GS\cdot s^{-1}$. **Fig. S8** shows the detailed calculating procedure for solving the distance automatically. First, we cut the interferogram into $K$ segments according to the period of the local comb, and number them $P_k$ ($k$=1~K). Then we use fast Fourier transform (FFT) on each segment of the waveform to obtain its frequency spectrum. Next, we search for the frequency $f_k$ with the highest signal-to-noise ratio (SNR) in the retrieved spectrum and calculate $\tau$ by using the **Eq. (13)**. According to the theoretical analysis in ***S1.1***, the sum of a pair of adjacent two $\tau_k$ can always be found at the place where the probe comb and the local comb, and the reference comb and the local comb interfere with each other to satisfy the **Eq. (1)**, corresponding to $\tau_1$, $\tau_2$ and $\tau_3$, $\tau_4$. Therefore, we can easily find $\tau_{m,1}$ & $\tau_{m+1,2}$ and $\tau_{n,3}$ & $\tau_{n+1,4}$ by querying $\tau_k$ in the two adjacent segments, where $m$ and $n$ are the number of segments they are located at. Then $N$ can be obtained by $N$ = n-m. Then we calculate the flight time $t_{tof}$ by **Eq. (2)**, and finally we can calculate the distance $L_D$ according to $L_D = c\cdot t_{tof}/2$.

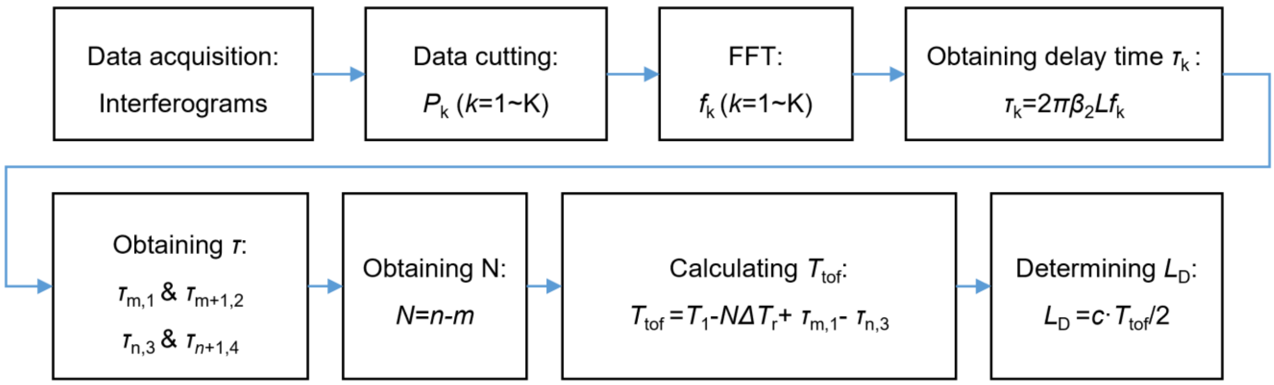


**Fig. S8. Detailed calculating procedure of distance solution.** The data process includes 8 steps: Data acquisition, Data cutting, FFT, Obtaining delay time $\tau_k$, Obtaining $\tau_{m,1}$ & $\tau_{m+1,2}$ and $\tau_{n,3}$ & $\tau_{n+1,4}$, Obtaining N, Calculating $t_{tof}$ and Determining $L_D$. All of the above processing steps can be done automatically by a personal computer.

### *S3.3 Comparison with traditional dual-comb TOF ranging*

Now we compare the DFT enhanced dual-comb ranging and traditional TOF ranging. The comparison is finished by using the same signal and local combs. We note that in traditional TOF method without DFT, for meeting the Nyquist sampling theorem, the repetition rate difference $\Delta f_{rep}$ and spectral bandwidth $\Delta\nu_{comb}$ of the two soliton pulse need to satisfy the following relations [2]:

$$\Delta f_{rep} < \frac{f_{r1} f_{r2}}{2\Delta \nu_{comb}} \tag{23}$$

The dual comb parameters used in the experiment are as follows: $f_{r1}$=24.465MHz, $f_{r2}$=24.55MHz, $\Delta\nu_{comb} \approx 0.855$ THz, $\Delta f_{rep} \approx 85$ kHz. Obviously, the above dual comb parameters do not satisfy **Eq. (23)**, resulting that no stable interference pattern will be generated, and only an intensifier peak due to too long relaxation time of the photodetector can be obtained. In this case, distance demodulation based on interference patterns is not applicable, and enhanced peak envelope fitting demodulation limits resolution. To achieve traditional dual-comb ranging under the existing repetition rate parameter, one solution is to reduce $\Delta f_{rep}$ to less than 351 Hz, and another is to compress $\Delta\nu_{comb}$ to less than 2.24 GHz. The former scheme greatly reduces the acquisition rate and accuracy while the latter scheme needs an optical filter with a bandwidth of < 0.028 nm, which will greatly reduce the SNR because the filter bandwidth is much smaller than the original spectral bandwidth. **Fig. S9** shows a schematic diagram of the ranging signal under three parameter profiles. **Fig. S9a** and **Fig. S9b** demonstrate that the traditional dual-comb TOF ranging mechanism requires a trade-off between detection resolution and detection rate. In our approach, this tradeoff is greatly weakened, as shown in **Fig. S9c**. Under the same light source parameters in **Fig. S9b**, the DFT-enhanced dual-comb ranging system can produce

resolved interference fringes stably at a high measurement frame rate of 85 kHz.

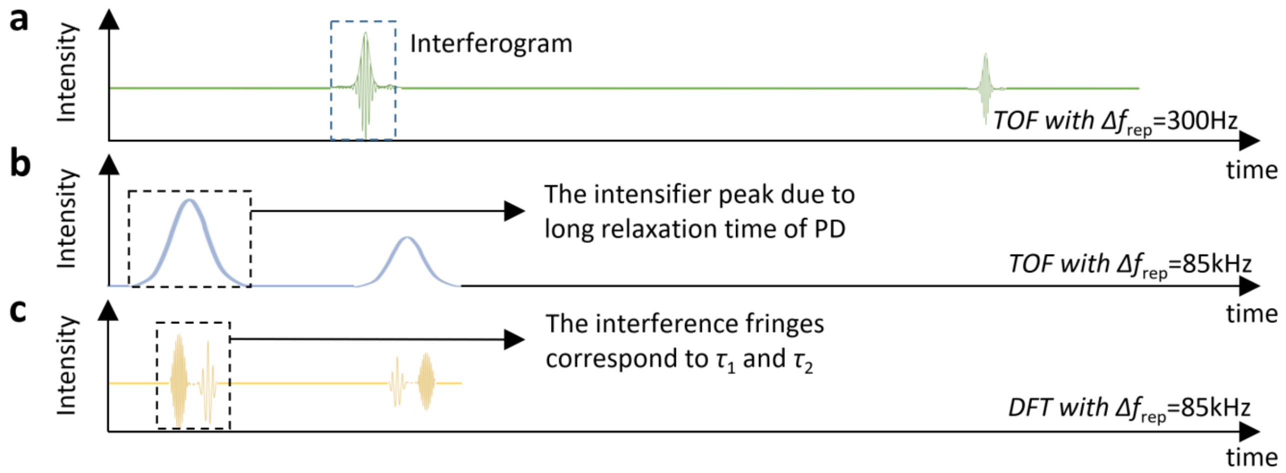


**Fig. S9. Comparison of measurement results between the two methods. a.** Traditional TOF method with $\Delta f_{\mathrm{rep}}$ = 300 Hz. **b.** Traditional TOF method with $\Delta f_{\mathrm{rep}}$ = 85 kHz. **c.** Our DFT enhanced dual-comb ranging with $\Delta f_{\mathrm{rep}}$ = 85 kHz.

### *S3.4 Ranging distance extension*

The non-ambiguity of a dual-comb ranging system can be significantly extended by leveraging the Vernier effect. This is achieved through the manual interchange of the roles between the two combs. In this discussion, we delve into the mechanism behind this. Consider the repetition rate of the signal comb is $f_{r1}$ and the repetition rate of this local comb is $f_{r2}$, a distance can be written as

$$L = \frac{c}{2}(MT_1 + \Delta t_1) \tag{24}$$

Here $T_1 = 1/f_{r1}$ is the period of the comb #1. $M$ is an integer. If $M$ =0, $L$ is in the non-ambiguity region of comb#1. However, when the repetition rate of the signal comb is $f_{r2}$, and the repetition rate of this local comb is $f_{r1}$, this distance is

$$L = \frac{c}{2}(NT_2 + \Delta t_2) \tag{25}$$

Here $T_2 = 1/f_{r2}$ is the period of the comb #2. $N$ is an integer. If $n$ =0, $L$ is in the non-ambiguity region of comb#2. In above equations, we call $\Delta t_1$ and $\Delta t_2$ 'wrapped times', $\Delta t_1 \le T_1$, $\Delta t_2 \le T_2$. For a given distance $L$, $MT_1 + \Delta t_1 = NT_2 + \Delta t_2$. Commonly in a dual comb ranging system, difference between $T_1$ and $T_2$ is tiny, for measuring a fixed $L$, $M = N$ [s11]. Therefore, we can obtain:

$$M = \frac{\Delta t_2 - \Delta t_1}{T_1 - T_2} \tag{26}$$

Therefore, the given distance $L$ can also be written as:

$$L = \frac{c}{2}\left(\frac{\Delta t_2 - \Delta t_1}{T_1 - T_2} T_1 + \Delta t_1\right) = \frac{c}{2}\left(\frac{\Delta t_2 T_1 - \Delta t_1 T_2}{T_1 - T_2}\right) \tag{27}$$

We can regard this equation as a binary function $L(\Delta t_1, \Delta t_2)$. When $\Delta t_2 = T_2$ and $\Delta t_1 = 0$, L reaches the maximum value:

$$L_{\max} = \frac{c}{2}\left(\frac{T_2 T_1}{T_1 - T_2}\right) = \frac{c}{2\Delta f_{rep}} \tag{28}$$

Relatively, when using using one single comb, one can easily know the maximum measurable distance is $c/2\ f_{rep}$. Typically, $\Delta f_{rep} << f_{rep}$, so that exchanging the roles of dual comb can extend the maximum measurable distance. Correspondingly, the use of double comb swapping operation will also slow down the frame rate.

Here we provide more discussions about the distance extension based on dual-comb vernier scheme. Since the repetition rate of the signal comb ($f_{r1}$) used in the experiment is 24.465 MHz, the range ambiguity $R=c/2f_{r1}$ is ~6.13 m in free space. Thanks to the Vernier effect [s12], a new range ambiguity $R'=c/2n\Delta f_{rep}$ of ~1.765 km can be achieved by simply exchanging the roles of signal and local combs. We measure the distance without exchanging and with exchanging the dual combs respectively, with a measurement step of 1 m. As shown in **Fig. S10a**, when the to-measure distance is larger than 8 m, the measured result will alias if we don't exchange the dual combs. However, in the case that we exchange the dual combs, we can obtain the correct distance. In the experiment, we use a fixed fiber collimator (LBTEK TAD-11) to collimate & launch the signal light to the free space with abeam diameter 2.0 mm. A movable target mirror is placed on an electrically controllable slide guides to precisely tune the distance (**Fig. S10b**).

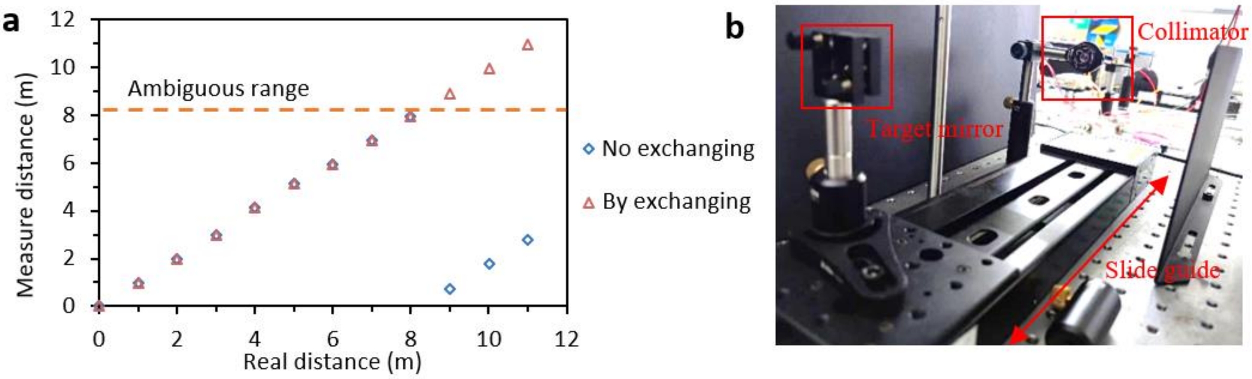


**Fig. S10. Extended distance by vernier effect. a.** Comparison of the result measured with/without exchanging. **b.** Photos of ranging devices.

### *S3.5 Ranging error analysis*

According to **Eq. (22)**, the ranging distance is proportional to the time of flight of the pulse. Therefore, the uncertainty of ranging can be obtained from the uncertainty of TOF. According to **Eq. (2)**, the TOF can be written as $t_{\mathrm{tof}} = NT_2\text{-}(N\text{-}1)T_1+\tau_1\text{-}\tau_3$. Since $N$ is an integer, the uncertainty of pulse time of flight depends mainly on the pulse period jitter of the light source and the fringe demodulation error of the system. In our method, the uncertainty of the measured $t_{tof}$ comes from two aspects: 1) the instability of $T_1$ and $T_2$; 2) the instability of the fiber system. Generally, the instability of the dual comb source plays the major role. We assume the instability of $T_1$ is $\theta_1$, while the instability of $T_2$ is $\theta_2$. Here could be a positive number or a negative number. Such an error will also influence $\tau_1$ and $\tau_3$, which indicates two pulse displacements. Therefore, the measured $t_{tof}$ could be written in $t_{\mathrm{tof}} = NT_2+\theta_2\text{-}(N\text{-}1)T_1+\theta_1+\tau_1\text{-}\tau_3+(\theta_1+\theta_2)$. We obtain the maximum measured temporal error is $2\theta_1+2\theta_2$, the maximum measured distance error is $c(2\theta_1+2\theta_2)$. For single shot measurement, frequency instabilities of both the two laser combs are smaller than 0.5 Hz (see **Fig. S5**) in each sampling period (11.76 μs). Referring the repetition rates of the two combs are 24.465 MHz and 24.55 MHz, $\theta_1 < 0.835$ fs, while $\theta_2 < 0.83$ fs. As a result, the comb instability limited error in ranging experiment is smaller than 250 nm. On the other hand, we discuss the averaging effect. After averaging, frequency uncertainty of the comb repetition is on $10^{-10}$ level (< 5 mHz), in this case either $\theta_1$ or $\theta_1$ is smaller than $5\times10^{-18}$ s. As a result, the dual comb uncertainty limited measurement error is < 1.5 nm. **Table S1** compares the dual comb limited precision in principle and the real measured precision in our measurement. Our ranging system doesn't reach the theoretical limitation, since there are random fluctuations in the fiber system.

**Table S1. Performance comparison: ranging accuracy**

| | **Single shot** | **After averaging** |
|---|---|---|
| **Limited by the dual comb instability in principle** | 250 nm | 1.5 nm |
| **Experimentally measured** | 262 nm | 2.8 nm |

***S3.6 Optical power and measurable distance***

In dual comb ranging, the reflected optical power critically influences the Signal-to-Noise Ratio (SNR). The factors that typically constrain the received optical power include laser power, diffuse reflection loss, the focal length and numerical aperture of the collimator, and the receiver's caliber,

among others. For a LIDAR system, assessing the reflected power across different distances and evaluating the necessity of an optical amplifier are essential.

Moreover, the dominant types of reflection vary across different ranging scenarios. In measurements of spatial distance or surface morphology, specular reflection predominantly contributes to strong reflectivity. Conversely, for objects like fan blades or drones (lacking cube-corner prisms), diffuse reflection is more significant. Our tests on received optical power in these scenarios are illustrated in **Fig. S11a** and **S11b**.

**Fig. S11c** presents the measured received power ratio using our dual comb ranging system to detect a UAV target, both with and without a cube-corner prism. The two curves represent the actual received optical power from a diffuse reflection target and a specular reflection target, respectively. In our experiments, we set the transmit laser power at 14 dBm (or 25.12 mW). For both diffuse and specular targets, the received power diminishes exponentially with increasing distance, primarily due to light absorption and divergence in the air. The minimum receivable power is -50 dBm, indicating that with a 14 dBm laser power, the maximum measurable distance for a diffuse object is approximately 81 m, whereas for a specular target, it exceeds 10 km.

One strategy to extend the measurable distance involves boosting the probe comb's sent-out power. **Fig. S11d** demonstrates this approach. Under conditions of diffuse reflection, the received optical power linearly escalates with an increase in transmitted optical power at a constant distance of 7 m. We estimate that at an optical power of 30 dBm (or 1 W) for the laser comb, our system can effectively detect a diffuse reflection target from 1 km away.

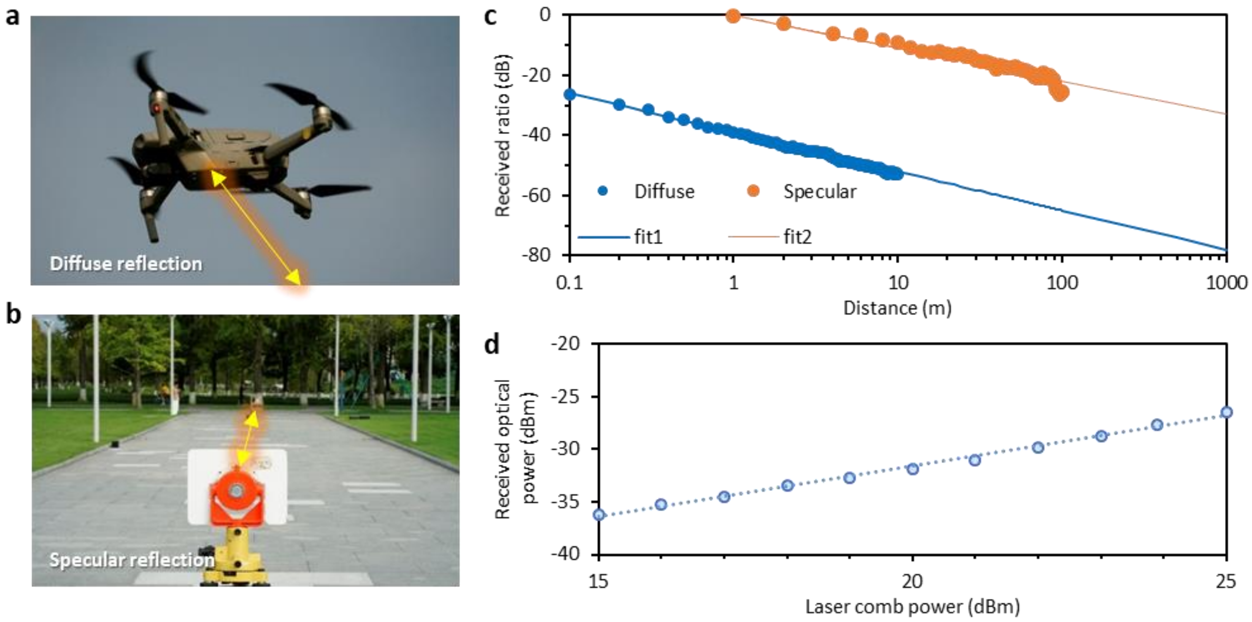


**Fig. S11. Discussion of the measurable distance. a & b.** Ranging scenarios, the target demonstrates

diffuse reflection or specular reflection. **c.** Received ratio in power, for diffuse and specular reflection. **d.** Received optical power scales with the sent-out laser comb power.

### *S3.7 System capacity and reliability*

In this discussion, we address the aspects of capacity, compatibility, and long-term stability of our devices, which are significant for the functioning of embedded systems. Initially, we introduce a dual comb module based on mode-locking fiber laser technology. This module can be efficiently packaged in a compact box within 20×15×10 $cm^3$ and weighing less than 1 kg, as illustrated in **Fig. S12a**. Within this module, we have seamlessly integrated a power supply, a 980 nm pump laser, two sets of mode-locked lasers, and two feedback stabilizers. The internal architecture is showcased in **Fig. S12b**, displaying the thoughtful separation between the optical and electrical components. The design incorporates two output ports intended for emitting two laser combs with distinct repetition rates. Concurrently, the system benefits from temperature control and vibration reduction features. Thanks to a vibration isolation treatment, each fiber laser comb can be easily connected and used (plug and play) with good stability. As a result, intensity uncertainty of each laser comb is maintained below 0.1% over a 48-hour period (as shown in **Fig. S12c**).

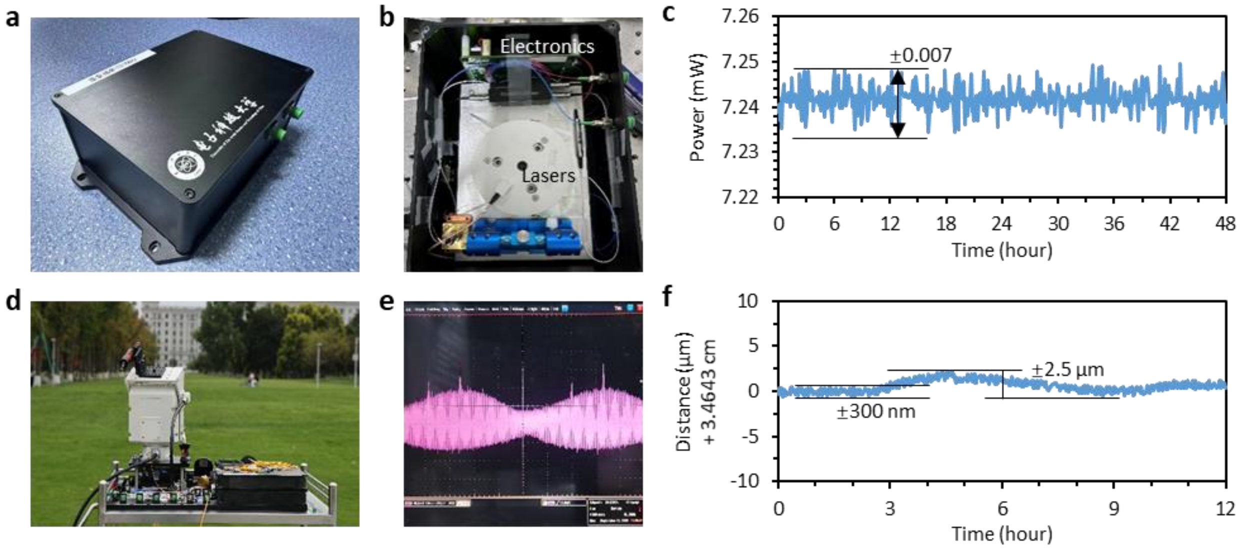


**Fig. S12. Capacity and reliability of the system. a.** Packaged device of our mode locked fiber laser based dual comb (20×15×10 $cm^3$). **b.** Internal architecture of the dual comb device. **c.** Long-term stability of the laser comb output. **d.** Picture of the DFT based dual-comb ranging system prototype. **e.** Data collection in an oscilloscope. **f.** Long-term stability for ranging a fixed target.

**Fig. S12d** reveals the prototype of our ranging system. This system extends beyond the laser comb source module to include a Pan-tilt, a transmitter & receiver, a signal processor, and a dispersive

delay component. All components are designed to fit within a platform having overall dimensions of 60×40×30 $cm^3$. To effectively visualize the outcomes of dual comb ranging, the utilization of an external oscilloscope is indispensable. **In Fig. S12e**, we demonstrate the scenario of data collection using an oscilloscope in a single frame. Concluding our assessment, we examine the long-term reliability of our ranging system. Conducted in a laboratory setting, we target a static object (mirror, 3.4643 m away from the transmitter) for a continuous duration of 12 hours, during which our system diligently records the measured distance data (illustrated in Fig. **S12f**). This testing confirms that the ranging error remains below $10^{-5}$. We identify that the system's inherent uncertainty stands at ±300 nm, while the target fluctuation is recorded at ±2.5 μm.

### *S3.8 Performance comparison*

**Table S2** compares key performances of recent ranging systems, including FCMW ranging, conventional dual-comb ranging, dual-comb ranging with the TPFC and the current DFT-based dual-comb ranging (this work).

**Table S2. Performance comparison of distinct ranging methods**

| Technique | Single shot accuracy | Distance | Speed |
|---|---|---|---|
| FMCW Ranging [s13] | 30 μm | NG | 1 Hz |
| FMCW Ranging [s14] | 115 μm | NG | 9.6 Hz |
| EOS-TD-Based TOF [s15] | 24 nm | 6 mm | 250 MHz |
| Microcomb DPI [s16] | 5.6 μm | 1.1 km | 35 kHz |
| Dual-microcomb TOF [s3] | 60 μm | 0.15 cm | ~1 GHz |
| Dual fiber comb TOF [s12] | 3 μm | 1.5 m | 5.19 kHz |
| Dual Ti: Sapph comb TOF [s17] | 510 μm | 29 cm | 220 kHz |
| Dual-comb with TPFC [s18] | 125 μm | 75 cm | 40 Hz |
| **This work** | **262 nm** | **1.7 km** | **85 kHz** |

NG: not given.

## Supplementary reference

[s1] Suh M-G, Vahala K J. Soliton microcomb range measurement[J]. Science, 2018, 359(6378): 884–887.

[s2] Zhu Z, Wu G. Dual-Comb Ranging[J]. Engineering, 2018, 4(6): 772–778.

[s3] Trocha P, Karpov M, Ganin D, et al. Ultrafast optical ranging using microresonator soliton

frequency combs[J]. Science, 2018, 359(6378): 887–891.
[s4] Zhou Y, Chan J C K, Jalali B. A Unified Framework for Photonic Time-Stretch Systems[J]. Laser & Photonics Reviews, 2022, 16(8): 2100524.
[s5] Mahjoubfar A, Churkin D V, Barland S, et al. Time stretch and its applications[J]. Nature Photonics, 2017, 11(6): 341–351.
[s6] Klauder J R. Path integrals and stationary-phase approximations[J]. Physical Review D, 1979, 19(8): 2349–2356.
[s7] Solli D R, Chou J, Jalali B. Amplified wavelength–time transformation for real-time spectroscopy[J]. Nature Photonics, 2008, 2(1): 48–51.
[s8] Herink G, Kurtz F, Jalali B, et al. Real-time spectral interferometry probes the internal dynamics of femtosecond soliton molecules[J]. Science, 2017, 356(6333): 50–54.
[s9] Li B, Xing J, Kwon D, et al. Bidirectional mode-locked all-normal dispersion fiber laser[J]. Optica, 2020, 7(8): 961.
[s10] Prakash N, Huang S-W, Li B. Relative timing jitter in a counterpropagating all-normal dispersion dual-comb fiber laser[J]. Optica, 2022, 9(7): 717.
[s11] Zhang H, Wei H, Wu X, et al. Absolute distance measurement by dual-comb nonlinear asynchronous optical sampling[J]. Optics Express, 2014, 22(6): 6597.
[s12] Coddington I, Swann W C, Nenadovic L, et al. Rapid and precise absolute distance measurements at long range[J]. Nature Photonics, 2009, 3(6): 351–356.
[s13] Bridger Photonics datasheets (https://www.bridgerphotonics.com/).
[s14] Luna Optical Backscatter Reflectometer (OBR) 4600 datasheet (https://lunainc.com).
[s15] Na Y, Jeon C-G, Ahn C, et al. Ultrafast, sub-nanometre-precision and multifunctional time-of-flight detection[J]. Nature Photonics, 2020, 14(6): 355–360.
[s16] Wang J, Lu Z, Wang W, et al. Long-distance ranging with high precision using a soliton microcomb[J]. Photonics Research, 2020, 8(12): 1964.
[s17] Mitchell T, Sun J, Reid D T. Dynamic measurements at up to 130-kHz sampling rates using Ti:sapphire dual-comb distance metrology[J]. Optics Express, 2021, 29(25): 42119.
[s18] Caldwell E D, Sinclair L C, Newbury N R, et al. The time-programmable frequency comb and its use in quantum-limited ranging[J]. Nature, 2022, 610(7933): 667-673.
